# Association of a variant in the regulatory region of NADPH oxidase 4 gene and metabolic syndrome in patients with chronic hepatitis C

**DOI:** 10.1186/s40001-015-0136-2

**Published:** 2015-03-28

**Authors:** Erika Rabelo Forte de Siqueira, Luciano Beltrao Pereira, Jose Tadeu Stefano, Thiago Patente, Ana Mercedes Cavaleiro, Luydson Richardson Silva Vasconcelos, Rodrigo Feliciano Carmo, Leila Maria Moreira Beltrao Pereira, Flair Jose Carrilho, Maria Lucia Corrêa-Giannella, Claudia P Oliveira

**Affiliations:** Department of Gastroenterology (LIM-07), School of Medicine, University of São Paulo, Av. Dr Arnaldo, 455, 3° Andar, #3115, Cep.: 1246903 São Paulo, Brazil; Laboratory for Cellular and Molecular Endocrinology - LIM-25, School of Medicine, University of São Paulo, Av. Dr Arnaldo, 455, 3° Andar, #3115, São Paulo, SP Brazil; Department of Gastroenterology, School of Medicine, University of Pernambuco, Av. Professor Morais Rego, 1235 Pernambuco, Brazil; Liver Institute of Pernambuco, Arnóbio Marques Street, 282 Recife, Pernambuco Brazil; College of Pharmaceutical Sciences, Federal University of São Francisco Valley, Avenida José de Sá Maniçoba - Centro, Petrolina, PE Brazil; NUCEL-NETCEM Cell and Molecular Therapy Center, School of Medicine, University of São Paulo, Av. Dr Arnaldo, 455, 3° Andar, #3115, São Paulo, SP Brazil

**Keywords:** Hepatitis C, NADPH oxidase 4 polymorphisms, Metabolic syndrome

## Abstract

**Background:**

Given the important contribution of the nicotinamide adenine dinucleotide phosphate (NADPH) oxidase system to the generation of reactive oxygen species induced by hepatitis C virus (HCV), we investigated two single nucleotide polymorphisms (SNPs) in the putative regulatory region of the genes encoding NADPH oxidase 4 catalytic subunit (*NOX4*) and its regulatory subunit p22phox (*CYBA*) and their relation with metabolic and histological variables in patients with HCV.

**Methods:**

One hundred seventy eight naïve HCV patients (49.3% male; 65% HCV genotype 1) with positive HCV RNA were genotyped using specific primers and fluorescent-labeled probes for SNPs rs3017887 in *NOX4* and −675 T → A in *CYBA*.

**Results:**

No association was found between the genotype frequencies of *NOX4* and *CYBA* SNPs and inflammation scores or fibrosis stages in the overall population. The presence of the CA + AA genotypes of the *NOX4* SNP was nominally associated with a lower alanine aminotransferase (ALT) concentration in the male population (CA + AA = 72.23 ± 6.34 U/L *versus* CC = 100.22 ± 9.85; mean ± SEM; *P* = 0.05). The TT genotype of the *CYBA* SNP was also nominally associated with a lower ALT concentration in the male population (TT = 84.01 ± 6.77 U/L *versus* TA + AA = 109.67 ± 18.37 U/L; mean ± SEM; *P* = 0.047). The minor A-allele of the *NOX4* SNP was inversely associated with the frequency of metabolic syndrome (MS) in the male population (odds ratio (OR): 0.15; 95% confidence interval (CI): 0.03 to 0.79; *P =* 0.025).

**Conclusions:**

The results suggest that the evaluated *NOX4* and *CYBA* SNPs are not direct genetic determinants of fibrosis in HCV patients, but nevertheless *NOX4* rs3017887 SNP could indirectly influence fibrosis susceptibility due to its inverse association with MS in male patients.

## Background

Chronic infection with hepatitis C virus (HCV) affects approximately 170 million people worldwide, and it is associated with increased morbidity and mortality secondary to cirrhosis and hepatocarcinoma (HCC) [1]. In Brazil, the national survey of viral hepatitis has shown an overall prevalence of positive HCV antibody of 1.38% [2]. The viral chronic infection stimulates a general tissue response, which includes oxidative stress as one of the pathogenic feature. HCV proteins stimulate the generation of reactive oxygen species (ROS) in infected tissue [3-6], and different cell types (hepatocyte and nonhepatocyte) contribute to hepatic oxidative stress in this condition [7].

It has been already demonstrated that HCV increases mitochondrial ROS production following mobilization of intracellular calcium. Another ROS source in HCV infection are nicotinamide adenine dinucleotide phosphate (NADPH) oxidase (NOX) proteins [7], membrane-associate proteins that transfer electrons across membranes and catalyze the NADPH-dependent reduction of O_2_ to O_2_^−^. Not only NOX2, the prototype of NADPH oxidase family, is expressed in the liver (hepatocytes, Kupffer cells, quiescent and activated hepatic stellate cells (HSC), and endothelial cells), but also the isoforms NOX1 and NOX4 [8], like NOX2, require association with another membrane-associate protein, subunit p22phox, encoded by the *CYBA* gene [9]. HCV was shown to elicit a persistent augment not only of NOX1 and NOX4 in hepatocytes *in vitro* and in the human liver but also of p22phox and other components of the NADPH oxidase system [10].

Thus, given the important contribution of the NADPH oxidase system to the oxidative stress induced by HCV infection, the aim of the present study was to evaluate association of two single nucleotide polymorphisms (SNPs) in the genes encoding NOX4 and p22phox (*CYBA*) with metabolic and histological variables in patients with chronic hepatitis C. *NOX4* rs3017887 was chosen because it was the most informative SNP (able to capture 12 out of 124 SNPs with minor allele frequency of at least 0.05) from a haplotype block comprised of four SNPs possibly associated with oxidative burden [11]. *CYBA −*675 T → A (unregistered) was chosen because it was previously shown to be functional, with the T allele associated with a higher phagocytic NADPH-dependent superoxide production [12].

## Methods

### Patients

We studied 178 patients with chronic hepatitis C infection (CHC) from the Northeast of Brazil. All patients enrolled had tested positive for anti-HCV antibodies (third-generation enzyme immunoassay, Abbott Laboratories, Chicago, USA) and HCV-RNA (RT-PCR, Roche Cobas Amplicor 2.0, Roche Diagnostics, Basel, Switzerland). All studied patients were naïve to anti-HCV therapy and did not present severe leucopenia, anemia, or plaquetopenia. The HCV genotype was determined by LiPA assay (Innolipa HCV II; Immunogenetics, Ghent, Belgium). All patients were enrolled at the Liver Institute of Pernambuco from February 2012 to October 2013.

This cross-sectional study was conducted according to the Helsinki declaration of 1975. Specific informed consent was obtained from all included patients, and the protocol was approved by the Internal Review Board of the University of Pernambuco - Brazil.

Other causes of liver disease were excluded after clinical, laboratory (viral serology, autoantibody titers, serum iron, ferritin and transferrin saturation, ceruloplasmin, copper and alpha1-antitrypsin concentrations), and image (hepatobiliary system ultrasound) examination. Patients who had a >100 g/week alcohol intake determined by a detailed personal history, questioning of family members, and an investigation of previous medical records were excluded.

### Study design and laboratory assays

Serum samples were collected at the time of liver biopsy and used to determine total cholesterol, low-density lipoprotein (LDL), high-density lipoprotein (HDL), triglycerides (Tg), alanine aminotransferase (ALT), aspartate aminotransferase (AST), gamma-glutamyl transferase (GGT), alkaline phosphatase (ALP), and fasting plasma glucose. Biochemical analyses were performed by standard methods using automated techniques (Cobas, Roche), and LDL concentrations were determined by the Friedwald equation [13]. HCV genotypes were determined by the INNO-LiPA HCV II kit (Versant, Bayer Diagnostics, Tarrytown, NY, USA) according to manufacturer’s instructions [14].

Diagnosis of type 2 diabetes mellitus (T2D), hypertension, and dyslipidemia was based on the criteria of the American Diabetes Association (Alexandria, VA, USA). The diagnosis of metabolic syndrome (MS) was conducted in accordance with the recommendations of the modified Adult Treatment Panel III (ATP III) criteria and required the presence of at least three of the following: a fasting plasma glucose ≥110 mg/dL, blood pressure ≥135/85 mmHg, Tg ≥150 mg/dL, HDL <40 mg/dL in men or <50 mg/dL in women, and waist circumference ≥ 102 cm in men or ≥88 cm in women [15].

### Genotype analysis

Two SNPs in the putative regulatory region of the genes encoding NADPH oxidase 4 catalytic subunit (*NOX4*) (rs3017887) and its regulatory subunit p22phox (*CYBA*) were selected based on previous studies [11,12]. The samples were genotyped by real-time PCR using fluorescent-labeled probes according to the procedures recommended by the manufacturer. *NOX4* rs3017887 was genotyped by assay type validation (C_15762095; Applied Biosystems, Foster City, USA), and *CYBA −*675 T **→** A (unregistered) was genotyped by PCR using specific primers (sense: 5′-GCGCTGGCTCACCAC-3′ and antisense: 5′-ACTGGGAAAGCACAGAATGCA-3′) and fluorescent-labeled probes (VIC: 5′-CCTCCCGAACCCAGG-3′ and FAM: 5′-CCTCCCGTACCCAGG-3′) (TaqMan, Applied Biosystems). Amplifications were performed on an Applied Biosystems Step One Plus Real-Time PCR System (30 s at 60°C, 10 min at 95°C, followed by 40 cycles of PCR (15 s at 95°C followed by 60 s at 60°C) and 30 s at 60°C). Genotyping success rates were 98% for both SNPs, and the distribution of genotypes was consistent with Hardy-Weinberg equilibrium for the two evaluated SNPs.

### Histological analysis

The liver tissue was fixed in 4% formaldehyde and processed for hematoxylin-eosin and Masson trichrome stains for histological analysis. Histological analyses were evaluated by only one pathologist who was unaware of the HCV genotype and of the patient’s clinical characteristics. Stages of fibrosis and grades of inflammation were scored according to METAVIR (F0: no fibrosis, F1: portal fibrosis without septa, F2: portal fibrosis with few septa, F3: numerous septa without cirrhosis, and F4: cirrhosis) [16]. Patients with histological evidence of non-alcoholic steatohepatitis (NASH) were excluded from the study.

### Statistical analysis

Results are expressed as mean ± SD except where stated otherwise. Continuous variables were log-transformed for the analysis when normal distribution was rejected by Shapiro-Wilk *W* test. Fisher’s chi square test, ANOVA, and ANCOVA were used for comparisons between groups. Odds ratios (OR) and 95% confidence intervals (CIs) were computed in the analysis for the minor alleles. Adjustments for clinical and biological variables were carried out by including them as covariates in the regressive model. Interaction between sex and genotype was assessed by including in the regression models (ANCOVA or logistic regression) a ‘crossed’ compound covariate (sex/genotype). The stratification by sex was then performed by nesting the genotype variable within the sex variable in the analysis model, resulting in the computation of statistical effects for men and women separately, and adjusted for multiple comparisons due to the stratification by sex. Moreover, as two independent SNPs were tested, a Bonferroni correction was applied: *P* ≤ 0.025 was considered significant, unless stated otherwise. The power to detect sex-specific associations of the SNPs with MS was approximately 40%, for an OR ≥1.5 and alpha = 0.05. Statistics were performed with the JMP software (SAS Institute Inc., Cary, NC, USA).

## Results

The clinical and biochemical variables according to the grade of fibrosis are demonstrated in Table 1. Patients with fibrosis grade F3/F4 were older than patients with fibrosis grade F1/F2; AST, GGT, and ALP concentrations increased with the severity of fibrosis. The frequency of MS was significantly higher in patients with fibrosis grade F3/F4, and the frequency of HCC was significantly higher in patients with fibrosis grade F4.

**Table 1:** **Clinical and biochemical variables according to the grade of fibrosis in patients with chronic hepatitis C**

	**F1**	**F2**	**F3**	**F4**	***P*** **value**
*N*	52	53	37	36	
Age^*^	53 ± 13	56 ± 9	60 ± 8	59 ± 11	0.05
Gender: M/F (%)	44/56	57/43	38/62	53/47	0.29
HCV genotype 1 (%)	57.7	56.6	78.4	75	0.18
HCV genotype 2 (%)	7.7	3.8	2.7	5.6	
HCV genotype 3 (%)	34.6	39.6	18.9	18.4	
Alcohol consumption (%)	58/27/15	49/32/19	67/19/14	61/11/28	0.38
Tabagism (%)	33/8/59	47/11/41	38/8/54	31/8/61	0.58
Type 2 diabetes (%)	15	26	16	19	0.49
Hypertension (%)	31	41	56	42	0.14
*Metabolic syndrome (%)*	*12*	*26*	*39*	*28*	*0.03*
*Hepatocarcinoma (%)*	*0*	*0*	*0*	*31*	*<0.001*
^*^BMI (kg/m^2^)	26.49 ± 3.93	26.17 ± 3.68	26.76 ± 5.43	26.67 ± 4.40	0.94
Waist circumference (cm)	91.29 ± 11.53	92.07 ± 9.14	94.32 ± 13.18	95.78 ± 11.45	0.28
TC (mg/dL)^*^	169 ± 34	169 ± 36	174 ± 55	151 ± 33	0.12
LDL (mg/dL)^*^	101 ± 33	100 ± 37	107 ± 50	82 ± 23	0.08
HDL (mg/dL)	52 ± 15	47 ± 16	47 ± 19	49 ± 20	0.23
Triglycerides (mg/dL)^*^	91 ± 31	139 ± 145	115 ± 50	90 ± 30	0.34
Fasting glucose (mg/dL)^*^	96 ± 36	101 ± 32	101 ± 33	102 ± 25	0.36
ALT (U/L)	70 ± 36	96 ± 59	86 ± 57	84 ± 54	0.22
*AST (U/L)*	*49 ± 25*	*71 ± 52*a	*84 ± 49*a	*101 ± 74*a,b	*<0.0001*
*GGT (U/L)*	*67 ± 50*	*98 ± 108*	*122 ± 86*a	*150 ± 123*a,b	*<0.0001*
*ALP (U/L)* ^*^	*78 ± 60*	*95 ± 65*	*92 ± 48*	*139 ± 80*a,b,c	*<0.0001*

Results are expressed as mean ± SD. For the continuous variables analysis, Shapiro-Wilk *W* test was tested, and variable failing in this test was log transformed. If even so the normality was not verified, nonparametric tests were applied for these variables (indicated by ^*^). ANOVA analysis or Kruskal-Wallis tests were performed, and the Tukey-Kramer HSD or Dunn test was used when significant differences were observed between the groups, being F1 (a), F2 (b), and F3 (c). Classes for ethnicity: Caucasoid, Negroid, and African descendant. Classes for alcohol: no drinking, <40 g/d, >40 g/d. Classes for smoke: ex-smoker, smoker, never-smoker. Abbreviations: GGT, gamma-glutamyl transferase; AST, aspartate aminotransferase; ALT, alanine aminotransferase; ALP, alkaline phosphatase; HDL, high-density lipoprotein; LDL, low-density lipoprotein; TC, total cholesterol; BMI, body mass index. Reference range: Glycemia ≤100 mg/dL; Triglycerides ≤150 mg/dL; HDL ≥40 mg/dL; LDL-C ≤130 mg/dL; TC ≤200 mg/dL; AST: Male 10 to 34 U/L, Female 10 to 36 U/L; ALT: Male 10 to 44 U/L, Female 10 to 36 U/L; GGT: Male 11 to 50 U/L, Female 7 to 32 U/L; ALP: Male 40 to 129 U/L, Female 35 to 104 U/L. *P* < 0.05 was considered significant (italicized data).

No associations were found between the genotype frequencies of *NOX4* rs3017887 (CC *versus* CA + AA) and *CYBA −*675 T → A (TT *versus* TA + AA) SNPs and inflammation scores (OR: 0.99; 95% CI: 0.45 to 2.14; *P =* 0.97 and OR: 0.99; 95% CI: 0.33 to 2.75; *P =* 0.99, for *NOX4* and *CYBA*, respectively) or fibrosis stages (Table 2) in the overall population and in the population stratified by sex or by HCV genotypes. In the male population, the CA + AA genotypes of the *NOX4* SNP presented a nominal (*P* > 0.025 to *P* ≤ 0.05) association with a lower ALT concentration (CA + AA: 72.23 ± 6.34 U/L *versus* CC: 100.22 ± 9.85 U/L; mean ± SEM; *P* = 0.05). The TT genotype of the *CYBA* SNP was also associated with a lower ALT concentration (TT = 84.01 ± 6.77 U/L *versus* TA + AA = 109.67 ± 18.37 U/L; mean ± SEM; *P* = 0.05) (Table 3).

**Table 2:** **Genotype frequencies by fibrosis grade in patients with chronic hepatitis C**

	**F1 and F2**	**F3 and F4**	**OR (95% CI)**	***P*** **value**
*NOX4* (rs3017887)	*N* = 105	*N* = 73	0.60 (0.22 to 1.59)	0.31
CC	0.532	0.588		
CA	0.457	0.372		
AA	0.011	0.040		
MAF	0.239	0.226		
*CYBA* (675 T → A)			0.29 (0.06 to 1.11)	0.09
TT	0.800	0.882		
TA	0.190	0.118		
AA	0.010	0.000		
MAF	0.105	0.059		

Genotype frequencies of *NOX4* rs3017887 and of *CYBA −*675 T → A (unregistered) single nucleotide polymorphisms by fibrosis grade in patients with chronic hepatitis C. Odds ratio (OR) and 95% confidence interval (95% CI) for the minor allele of the genotyped SNPs in a dominant model of logistic regression analysis adjusted for age, gender, metabolic syndrome status, triglycerides, plasmatic albumin concentrations, and HCV genotype. MAF, minor allele frequency (frequency of the rarer allele, A for both SNPs).

**Table 3:** **Alanine aminotransferase (ALT) concentrations in male patients with chronic hepatitis C**

	**ALT (U/L)**	***P*** **value**
*NOX4* (rs3017887)^a^		
CC	100.22 ± 9.85	0.05
CA/AA	72.23 ± 6.34	
*CYBA* (−675 T → A)^b^		
TT	84.01 ± 6.77	0.05
TA/AA	109.67 ± 18.37	

Alanine aminotransferase (ALT) concentrations in male patients with chronic hepatitis C according to the genotype of *NOX4* rs3017887 and *CYBA −*675 T → A single nucleotide polymorphisms. Results are mean ± SEM. ^a^ANCOVA adjusted for age, gender, metabolic syndrome status, gamma glutamyl transferase, and HCV genotype. ^b^ANCOVA adjusted for age, gender, metabolic syndrome status, systolic blood pressure, plasmatic albumin concentration, body mass index, and HCV genotype.

Table 4 shows the genotype frequencies of *NOX4* rs3017887 SNP according to the MS status in the population stratified by sex; we observed a lower frequency of MS in male patients carrying the genotypes CA + AA (CA + AA: 13.8% *versus* CC: 25.0%). This lower prevalence was confirmed in a dominant model of logistic regression analysis demonstrating a protective effect for the minor A-allele (OR: 0.15; 95% CI: 0.03 to 0.79; *P =* 0.025). No associations were found between the SNPs and the MS status when the population was stratified by HCV genotype.

**Table 4:** **Genotype frequencies of** ***NOX4*** **rs3017887 single nucleotide polymorphism by metabolic syndrome (MS) status**

***NOX4*** **(rs3017887)**	**Absence of MS**	**Presence of MS**	**OR (95% CI)**	***P*** **value**
Female	(*N* = 63)	(*N* = 25)	1.71 (0.48 to 6.00)	0.40
CC	0.540	0.476		
CA	0.440	0.524		
AA	0.020	0.000		
MAF	0.240	0.321		
Male	(*N* = 66)	(*N* = 17)	0.15 (0.03 to 0.79)	0.025
CC	0.545	0.714		
CA	0.436	0.286		
AA	0.018	0.000		
MAF	0.236	0.143		

Genotype frequencies of *NOX4* rs3017887 single nucleotide polymorphism by metabolic syndrome (MS) status in patients with chronic hepatitis C. Odds ratio (OR) and 95% confidence interval (95% CI) for the minor allele of rs3017887 SNP in a dominant model of logistic regression analysis adjusted for age, gender, type 2 diabetes status, body mass index and HCV genotype. MAF, minor allele frequency (frequency of the rarer allele, A for both SNPs).

## Discussion

The association between HCV and metabolic factors has been recognized as a two-way road. On the one hand, HCV infection is able to induce insulin resistance, steatosis, and diabetes; on the other hand, metabolic factors such as insulin resistance may modulate the clinical course of HCV infection, increasing the predisposition to hepatic fibrosis and to HCC [17]. Accordingly to these observations, MS was significantly more frequent in patients with F3/F4 in the present series. Interestingly, we found an association of the minor A-allele of *NOX4* rs3017887 with a lower prevalence of MS in this cohort of CHC patients, while no associations were observed between rs3017887 and inflammation scores or fibrosis stages.

We have no definitive explanation for the protective effect exerted by the A-allele of *NOX4* rs3017887 regarding MS, especially because this same SNP did not influence the frequency of MS in patients with non-alcoholic fatty liver disease (data not shown). One possibility is that this SNP manifests its effects only in presence of a stronger insult such as the viral infection; in other words, this may be a case of a gene (*NOX4* rs3017887)-environment (HCV) interaction: the SNP and HCV jointly influence the risk of developing MS, as already described for other conditions, for example, two SNPs in the *CYP1A1* gene that are associated with increased risk of HCC only in cigarette smoking subjects [18].

The modulation of the risk for MS by a variant in a gene encoding a protein from NADPH oxidase family is plausible because this pro-oxidant system is a major source of ROS in this metabolic condition [19]. It is noteworthy that no associations were found between *NOX4* rs3017887 and any of the components of MS alone, maybe because the SNP exerts a weak pleiotropic effect on each one of the MS traits that becomes noticeable only when the traits are considered together. The C-allele of rs3017887 was described in a haplotype block of four SNPs possibly associated with oxidative burden [11]; thus, it is feasible that the A-allele is associated with a lower oxidative stress. However, as far as we know, a direct functional repercussion of *NOX4* rs3017887 has never been demonstrated; and it is also possible that this SNP is not causative but a marker of another functional variant in linkage disequilibrium with it. Why the association was observed in a sex-specific manner remains to be further elucidated, but two of us (TP and MLCG) already observed a sex-specific association of other pathological condition with genes associated with the redox state [20].

Other positive findings of the present study were the nominal associations of the genotypes CA + AA of *NOX4* rs3017887 and of the genotype TT of *CYBA −*675 T → A with lower ALT concentrations. The association of the A-allele of rs3017887 is easier to understand since a lower necrosis activity could take place in the context of a lower oxidative burden [21]. However, the T-allele of *CYBA −*675 T → A has been previously associated with a higher NADPH-dependent superoxide production in peripheral monocytes and lymphocytes [12], which makes its association with lower ALT concentrations unexpected. Nevertheless, the functional effect of a genomic variant in one cell type does not necessarily mean the same functional effect in another cell type [22], and the functional repercussions of *CYBA −*675 T → A in hepatocytes has never been explored. Interestingly, CYBA was recently identified as a host factor implicated in HCV entry in hepatocytes, but the mechanisms underlying its participation in viral infection are currently unknown [23]. We speculate that CYBA not only takes part in HCV entry, but variations in its expression level could also modulate hepatocyte responses to HCV infection, which could explain, for instance, a greater or lesser necrosis activity in different individuals with distinct genetic variants.

## Conclusions

The results of the present study should be interpreted in the context of limitations imposed by the small number of patients, such as the low statistical power. Our findings suggest that the evaluated *NOX4* and *CYBA* SNPs are not direct genetic determinants of inflammation or fibrosis in HCV patients. However, in male patients with CHC, *NOX4* rs3017887 SNP could indirectly influence fibrosis susceptibility due to its inverse association with MS in male patients.
